# Letter Writing

**Published:** 1873-03

**Authors:** 


### LETTER WRITING.

New York, Thursday, Sept. 12, 1872. Mr. Robert Stephen. *Dear Sir:* Your letter, dated August 31st, 1872, enclosing ten dollars, is just received, etc., etc.

One thousand dollars would be saved every year, eleven hundred and nineteen annoyances; sixteen hundred hours of trouble; two thousand two hundred tear drops; five thousand and sixty suspicions; eight thousand sinkings of the heart; eighty-six grits of the teeth, and two hundred and ten bitter curses, if, for one year, every person who writes a letter should truthfully embody the points contained in the first three lines above.

If every letter received were replied to on the spot, the day and the hour, the answer would be easier; every day it is deferred makes it more difficult; you come to the work with less and less spirit; every time you think of that unanswered letter there is the accompaniment of a certain degree of disagreeableness, with a growing inability to form a satisfactory excuse, involving a longer explanation and greater care in framing one which will steer clear of a fib. How ardently are the answers to letters looked for sometimes—love letters for example! What terrible palpitations; what distressing sinkings of the heart; what troublous surmisings; what paralysing misgivings would a prompt reply prevent!

The place and date being stated informs the correspondent where to direct his answer; gives an idea of how long it would require to reach its destination. and tells him the day of the week on which it was written, preventing the trouble of hunting up an almanac and making various countings and computations.

If a man sends you money in a letter, he very naturally has some solicitude about its safety, as the loss would be all his own, and it is but fair that you should put an end to that solicitude as soon as possible; the loss of ten dollars may not be much to you; it may be the savings of months of daily labor to the one who sends it.

Thousands of letters are written by persons in urgent need of money, in distressing need, written to brothers, parents, children; affection promptly responds, anxious that it should reach its destination at the earliest hour possible, troubled in mind, it may be, in the fear of miscarriage, and distressed with conjectures of the misery which might follow if it did not go safely and promptly. And yet there are thousands of money letters received, the recipients, all regardless of their duty, and relieved of their load, wickedly allow days, and weeks even, to pass without any acknowledgment whatever.

Sometimes a world of trouble is occasioned by the want of a date or designation in a letter. There is many a man who would give a great deal—a hundred or a thousand or ten thousand dollars—to know the date of a memorandum made. A world of trouble would be prevented if persons would accustom themselves whenever they take up a pen to write a line to anybody, or make a memorandum about anything, to commence as above, nor can there be any reason why all letters should not begin thus; it would save paper, save time, and concentrate in one line a very large amount of information satisfactory, valuable, indispensable.

Persons may afford themselves no inconsiderable satisfaction throughout all of after-life by the little expedient of writing their name in a newly purchased book, the date, the price, as: John Smith, Sept. 12, 1872, Thursday, Rome. $1.50. Mary Brown, Geneva, Aug. 18, 1872, Wednesday, five francs. To the writer such memoranda have been the source of a wonderful amount of satisfaction. Comparing two books it was brought to light that on a certain day and month and year he was at a certain place; twenty-five years later, on the same day of the month, he was in the city of Glasgow, and in the cottage where Burns was born—no important thing for him to know, and yet there was a certain degree of pleasure in noticing the coincidence; a lonely, wandering, unconcerned, harum scarum "bach" in the first place; in the second place going over the same ground with four strapping, grown-up children; and so full of fun! and at the end of another twenty-five years to be treading on other than the

"Banks and braes of Bonny Doon,"

fresher and fairer far, where the flowers are fadeless and the trees bloom immortal. Reader, in due time may you be along.

